# Associations of street-view greenspace exposure with cardiovascular health (Life’s Essential 8) among women in midlife

**DOI:** 10.1186/s13293-025-00718-3

**Published:** 2025-07-01

**Authors:** Sheryl L. Rifas-Shiman, Li Yi, Izzuddin M. Aris, Pi-I Debby Lin, Marie-France Hivert, Jorge E. Chavarro, Esra Suel, Peter James, Emily Oken

**Affiliations:** 1 Division of Chronic Disease Research Across the Lifecourse, Department of Population Medicine, Harvard Medical School and Harvard Pilgrim Health Care Institute, Boston, MA USA; 2 Diabetes Unit, Department of Medicine, Massachusetts General Hospital, Boston, MA USA; 3 Department of Nutrition, Harvard T.H. Chan School of Public Health, Boston, MA USA; 4 Centre for Advanced Spatial Analysis, University College London, London, UK; 5 Department of Environmental Health, Harvard T.H. Chan School of Public Health, Boston, MA USA; 6 Department of Public Health Sciences, University of California Davis School of Medicine, Davis, CA USA

**Keywords:** Green space, Street-view imagery, Deep learning algorithms, Cardiovascular health, Life’s essential 8, Females, Midlife

## Abstract

**Background:**

Many women experience suboptimal cardiovascular health (CVH) during midlife. Greenspace exposure has been inversely associated with cardiovascular disease because it may reduce harmful environmental exposures and promote healthy behaviors. Most prior studies used satellite-based rather than ground-level exposures and did not examine overall CVH.

**Methods:**

We performed a longitudinal analysis of women in the Project Viva cohort based in Eastern Massachusetts. We applied deep learning algorithms to Google Street View images to derive metrics of visible trees, grass, and other greenspace within 500 m of participant’s residential addresses in 2012–2016 (mean age 46 years). About five years later (mean age 51 years), participants completed questionnaires and research measurements including blood collection. We calculated CVH scores using Life’s Essential 8 (LE8) construct (0-100 points, higher = better), which includes four behavioral (diet, physical activity, sleep, and avoidance of smoking) and four biomedical measures (body mass index, blood pressure, blood lipids, and blood glucose). We used linear regression models adjusted for age and both individual- and neighborhood-level socioeconomic status.

**Results:**

Among 767 participants, 68% were non-Hispanic White, and 74% were college graduates. Mean (SD) CVH score was 72 (13) points. When including three greenspace components in the same model, higher % trees (per SD) was associated with higher overall CVH score (β = 2.4; 95% CI: 1.3, 3.5), as well as higher behavioral (β = 2.8; 95% CI: 1.4, 4.3) and biomedical (β = 2.8; 95% CI: 1.0, 4.7) sub-scores. Additionally, % other greenspace (per SD) was associated with better biomedical CVH scores (β = 2.2; 95% CI: 0.4, 3.9), whereas associations for % grass were non-significant. Higher % trees (per SD) was associated with higher scores for most individual CVH components, including diet (β = 2.1 points; 95% CI: 0.7, 3.4), physical activity (β = 4.0; 95% CI: 1.2, 6.9), sleep (β = 2.6; 95% CI: 0.9, 4.4), BMI (β = 5.8; 95% CI: 2.8, 8.8), and blood glucose (β = 2.2; 95% CI: 0.3, 4.2).

**Conclusions:**

Greater street-view greenspace exposure, especially visible trees in streetscapes, was associated with better CVH among midlife women. Increasing trees in neighborhoods may be a valuable public health strategy to improve multiple metrics of cardiovascular health.

**Supplementary Information:**

The online version contains supplementary material available at 10.1186/s13293-025-00718-3.

## Abstract (plain language summary)

Many women experience suboptimal cardiovascular health during midlife, increasing their risk of heart disease. Research suggests that exposure to greenspace, such as vegetation and trees, may benefit heart health, but most studies have relied on satellite images rather than street-level views. In this study, we used Google Street View images and artificial intelligence to measure visible greenspace around the homes of 767 women from the Project Viva study in Eastern Massachusetts. We assessed greenspace exposure from trees, grass, and other plants within 500 m of their homes between 2012 and 2016 and examined its association with cardiovascular health scores about five years later, accounting for individual and neighborhood socioeconomic status.

We evaluated cardiovascular health scores using the Life’s Essential 8 (LE8) construct, which includes behavioral factors (diet, physical activity, sleep, and smoking) and biomedical factors (body mass index, blood pressure, blood lipids, and blood glucose). Our results showed that women living in areas with more total greenspace—particularly more trees—had higher overall cardiovascular health scores. Living in an area with higher amounts of visible trees was associated with a healthier diet, better sleep, higher physical activity levels, smoking avoidance, and healthier body weight, blood pressure, and blood sugar levels.

These findings suggest that increasing trees in neighborhoods could support better cardiovascular health among women in midlife. Policies promoting urban tree planting may be a valuable public health strategy to improve heart health and overall well-being.

**Supplementary Information:**

The online version contains supplementary material available at 10.1186/s13293-025-00718-3.

## Highlights

We examined the associations of street-view greenspace exposure with cardiovascular health among midlife women in the Project Viva cohort.

We quantified greenspace exposure using deep learning models applied to Google Street View images, measuring the percentage of trees, grass, and other plants within view in images within 500 m of participants’ homes.

We assessed cardiovascular health using the Life’s Essential 8 (LE8) score, which includes behavioral (diet, physical activity, sleep, smoking) and biomedical (BMI, blood pressure, lipids, glucose) components.

We found that greater street-view greenspace, particularly trees, was associated with higher overall cardiovascular health scores, including healthier behaviors and biomedical measures.

Increasing trees in neighborhoods may be a valuable public health strategy to improve cardiovascular health among midlife women.

**Supplementary Information:**

The online version contains supplementary material available at 10.1186/s13293-025-00718-3.

## Background

Cardiovascular health (CVH) often declines during midlife, particularly among women, making it a crucial life stage for clinical and public health efforts. For example, Healthy People 2030 identified improving CVH among adults as a high public health priority that has remained stagnant recent years [1]. As defined by the American Heart Association [2], CVH encompasses both behavioral factors, such as diet and physical activity, and biomedical markers, including blood pressure and blood glucose levels. Midlife represents a particularly important transitional period for women’s cardiovascular health due to the onset of menopause and associated hormonal changes, which are linked to increased cardiovascular risk factors such as dyslipidemia, hypertension, and insulin resistance [3, 4, 5, 6]. As such, understanding how to optimize health during this life stage is critical for preventing disease and promoting well-being during and beyond the menopausal transition. Maintaining CVH in midlife is associated with a lower risk of cardiovascular disease, greater longevity, and improved quality of life [7].

Exposure to greenspace has been linked to positive cardiovascular outcomes, with evidence suggesting that individuals spending time in greener environments tend to have lower risks of obesity, better blood glucose control, and healthier blood pressure levels [8, 9, 10, 11, 12, 13]. These benefits may be attributed to both physiological and psychological pathways, including increased physical activity, improved sleep, and enhanced mental health [14, 15, 16]. Greenspace may also be associated with lower exposures to air pollution and noise, which are linked to worse CVH [17, 18].

Most studies assessing greenspace rely on satellite-derived metrics, such as normalized difference vegetation index (NDVI), which provide a generalized birds-eye overview of vegetation quantity [19]. While these measures are valuable for comparing greenness levels across large geographic areas, they are limited in their ability to differentiate between specific types of vegetation, such as trees and grass, which may have differential health effects, or to capture the greenery individuals actually see and interact with in their daily routines [20].

Street-level greenspace metrics may better capture this direct visual and experiential exposure. Recent studies have shown that specific components of street-level greenery are more strongly associated with psychological restoration, lower depression risk, and cardiovascular benefits than satellite-based indices, [21, 22] suggesting that the visual presence and type of greenspace may play a critical role in shaping health-relevant behaviors and perceptions.

Street-view-based methods have emerged as an alternative for exposure assessment, enabling a more precise evaluation of greenspace components [23, 24, 25, 26]. While prior research has explored the relationship between street-view greenspace and select aspects of CVH, there remains limited investigation into its association with overall CVH, especially among midlife women. A recent study from Project Viva examined these relationships among children, highlighting the potential impact of greenspace exposure on early-life cardiovascular health [21]. This study builds on that work by conducting a similar analysis focused on their mothers in midlife.

We hypothesized that higher levels of street-view trees would be associated with better CVH in this population. By employing a refined approach to greenspace assessment, this study aims to provide deeper insights into the role of environmental factors in shaping CVH among midlife women.

## Methods

### Study population

Study participants were women enrolled in the ongoing Project Viva cohort, which recruited pregnant women at their initial prenatal visits at a multispecialty group practice in urban/suburban Eastern Massachusetts [27, 28]. Eligibility criteria included enrollment before 22 weeks of gestation, the ability to communicate in English, plans to remain in the study area through delivery, and singleton gestation.

For this analysis, we used covariate data from the enrollment pregnancy (1999–2002), residential address data from the Year 13 follow-up visit (2012–2016, mean age 46 years), and outcome data from the Mid-Life Visit (2017–2021, mean age 51 years). Of 2,100 women with live births, 843 had any Mid-Life Visit data, and we calculated CVH scores for 820 women. The final analytic sample included 767 women with available street-view greenspace exposure data from the Year 13 visit. Compared with the 1333 women originally enrolled who did not provide exposure and outcome data for the current analysis (Additional File 1), those included were more likely to have a college education (74% v. 59%) and a household income >$70,000/year at enrollment (64% v. 59%). There were no substantial differences in race and ethnicity (9% v. 9% Hispanic), marital status (93% v. 91% married or cohabiting), or mean pre-pregnancy body mass index (BMI) (24.6 v. 25.0 kg/m^2^) or neighborhood median household income ($57,100 v. $56,000 /year) at baseline. Participants provided written informed consent at enrollment and subsequent study visits. The Institutional Review Board of the Harvard Pilgrim Health Care Institute approved all study procedures.

### Green space exposure

#### Street-View green space metrics

To assess greenspace exposure from street-view images, we utilized deep learning segmentation models. First, we created a street network grid at 100-meter intervals along the street network across all Core-based Statistical Areas (CBSA) in the United States. Next, we identified and collected Google Street View images from 2007 to 2020 for each grid point, capturing four directional images (facing up and down the street, as well as to each side) per location to obtain a comprehensive 360-degree view. We exclusively used images captured by Google’s Street View cars, which adhere to a uniform standard of camera quality and positioning. This uniformity is crucial for maintaining the integrity of the data and ensuring the accuracy of our analysis.

To process each image, we applied the Pyramid Scene Parsing Network (PSPNet) algorithm (https://github.com/hszhao/PSPNet), a deep learning segmentation model pre-trained on the ADE20K dataset (https://github.com/CSAILVision/sceneparsing/blob/master/objectInfo150.csv). PSPNet segmented images at the pixel level into various greenspace features, including trees, grass, and other greenspace (plants, fields, flowers). Although we did not independently evaluate the model’s performance in this study, PSPNet has been extensively validated, achieving 81.69% pixel-level accuracy and a mean intersection over union (IoU) of 44.94% when tested on the ADE20K validation dataset [29, 30]. The algorithm then estimated the percentage of pixels of each greenspace feature within the images, averaging these values across the four orientations to determine overall greenspace exposure at each location. To enhance spatial coverage, we aggregated the data to a 100-meter raster for every CBSA in the U.S., imputing missing values using the nearest available street-view data in time. We linked these 100-meter street-view greenspace metrics to geocoded participant addresses at the Year 13 visit to derive exposure metrics. Additionally, we applied a spatial averaging technique using a 500-meter moving window to calculate smoothed street-view greenspace exposure by summarizing the mean values of 100-meter raster with the respective distance window around each participant’s location. Overall, the street-view approach allowed us to quantify specific types of greenspace exposure at ground level, offering a more precise representation of the actual environment experienced by participants [21].

#### Satellite-Based NDVI

We also linked 30-meter Landsat-derived Normalized Difference Vegetation Index (NDVI) estimates (ranging from − 1 to 1) to participants’ addresses at the time of their Year 13 visit (2012–2016), using data from July of the corresponding visit year. We calculated NDVI estimates for a 270-meter buffer surrounding each address [21, 31]. We set negative NDVI values that represent water to zero to not downweigh water.

We selected buffer sizes based on prior literature and methodological considerations [32]. Given the uncertainty regarding the most relevant geographic area for assessing spatial exposures [33, 34], we explored multiple buffer sizes to assess greenspace. For satellite-based greenspace, we derived NDVI estimates using 90-meter, 270-meter, and 1230-meter buffers. For street-view-based greenspace, we assessed images within 100-meter, 500-meter, and 1000-meter buffers. We selected a 500-meter buffer for street-view exposures to represent a walkable area around the home and a 270-meter buffer for NDVI estimates, as it was closest to the 500-meter street-view metric. Exposures for both satellite-based and street-view-based greenspace metrics were highly correlated across buffer sizes, so the choice of buffer size likely had minimal impact on our results.

#### Cardiovascular health at midlife

Our primary outcome was cardiovascular health (CVH) scores assessed using the American Heart Association’s Life’s Essential 8 (LE8) construct [2]. LE8 includes eight components categorized into two domains: behavioral and biomedical. The behavioral domain consists of diet quality (Healthy Eating Index), physical activity (minutes of light, moderate, and vigorous activity), sleep health (duration), and smoking exposure. The biomedical domain includes measured BMI, blood pressure, blood lipids (non–HDL cholesterol), and glycemia (fasting glucose, hemoglobin A1c, or diabetes status). Details on the assessment of each LE8 metric and the calculation of component scores in Project Viva were published by Nichols et al., [35] following the approach described by Lloyd-Jones et al. [2].

We calculated scores for each of the eight components on a scale from 0 to 100, with higher scores indicating better CVH. We then averaged component scores to derive overall CVH score and behavioral and biomedical sub-scores. Our primary outcomes were the overall CVH score and the behavioral and biomedical sub-scores, while our secondary outcomes included each of the eight individual component scores.

For individuals with complete LE8 data, we calculated the overall score using all eight measured components. For those with incomplete data (< 8 components, *N* = 279), we applied a validated machine learning model, as described by Zheng et al., [36] to predict the overall CVH score based on CVH-related factors that were routinely collected in Project Viva (i.e., age, self-reported history of diabetes, hypertension, and hypercholesterolemia, BMI, BP, nicotine exposure, and physical activity) across all study visits. We noted strong correlations (ρ = 0.92) between observed and predicted overall CVH scores at the Mid-life Visit, which suggests high reliability of the predicted CVH scores.

### Covariates

#### Assessment of covariates

At enrollment, participants reported their date of birth, education, marital status, household income, and receipt of public assistance through interviews and questionnaires [27, 28]. In midlife, they also reported their race and ethnicity, which we categorized as Hispanic, non-Hispanic White, non-Hispanic Black, non-Hispanic Asian, or other. We considered race and ethnicity as social constructs rather than biological determinants of disease risk [37, 38]. We included these characteristics in our study as proxy measures of structural racism, which may have implications for CVH and may be linked to greenspace in their neighborhood. We calculated age at each study visit from date of birth.

#### Socioeconomic status covariate

A previous Project Viva manuscript describes the methodology for creating the socioeconomic status (SES) index in detail [39]. Briefly, we developed the SES index using both individual- and neighborhood-level metrics at enrollment associated with health outcomes. Individual-level factors included maternal education, marital status, household income, and receipt of public assistance at enrollment. Neighborhood-level metrics included median income and the percentage of residents living below the poverty line, based on participants’ residential addresses at enrollment, which we linked to 2000 census data.

To construct the SES index, we assigned weighted scores (0 = lowest SES, 3 = highest SES) to each metric: maternal education (no college = 0; some college = 1; college degree = 2; graduate degree = 3), marital status (not married/cohabiting = 0; married/cohabiting = 3), household income at enrollment (<$20,000 = 0; $20,000–40,000 = 1; $40,000–70,000 = 2; >$70,000 = 3), and receipt of public assistance (yes = 0; no = 3). We categorized neighborhood-level measures into quartiles: median neighborhood income (Q1 = 0; Q2 = 1; Q3 = 2; Q4 = 3) and percentage of residents living below the poverty line (Q4 = 0; Q3 = 1; Q2 = 2; Q1 = 3).

We then conducted an unsupervised principal components analysis, retaining the first factor as the SES index score, which explained 68.6% of the variability in the original components. This factor represents a continuous, normally distributed composite SES score that reflects the natural intercorrelations among the six components, with higher values indicating higher SES.

### Statistical analysis

We used multivariable multilevel linear regression to examine the prospective associations of Year 13 visit street-view greenspace exposure with midlife CVH score measured approximately five years later. Given the hierarchical structure of the data, we specified a random intercept for census tract to account for potential clustering of individuals within census tracts. Our primary exposures included % total greenspace as well as its three components (% trees, % grass, % other greenspace) measured at the Year 13 visit. We included the three greenspace components (% trees, % grass, % other greenspace) in the same model to examine the independent associations of each of the three greenspace components with CVH. Additionally, we assessed satellite-based NDVI as a secondary exposure. We analyzed % total greenspace and NDVI separately, with each model excluding the three greenspace components. To enable direct comparisons across greenspace measures, we standardized all GSV and NDVI exposure metrics to z-scores, allowing interpretation of effects per standard deviation (SD) increase.

Our primary outcomes included the overall CVH score, as well as behavioral and biomedical sub-scores, measured at the Mid-Life Visit. We also evaluated the eight individual CVH component scores as secondary outcomes. We conducted unadjusted analyses (Model 1), and analyses adjusted for age and socioeconomic status (SES) index score (Model 2), incorporating both individual- and neighborhood-level metrics. In Model 3, we further adjusted for race and ethnicity, which we considered a social construct rather than a biological variable, as these factors may be associated with residential location and lifetime health outcomes [40].

In a sensitivity analysis, we examined street-view exposures additionally adjusted for satellite-based NDVI to examine the associations contributed by street-view metrics independent of satellite-based measures.

To address missing covariate data, we applied multiple imputation by chained equations (20 imputations). We performed all analyses using SAS Enterprise Guide, Version 8.3 (SAS Institute, Cary, NC).

## Results

Among 767 participants, 68% reported being non-Hispanic White, and 74% were college graduates. The mean (SD) age at CVH outcome assessment was 51.0 (5.1) years. Mean (SD) CVH scores were 72.2 (13.9) points overall, 72.6 (18.1) for the behavioral domain, and 68.8 (22.7) for the biomedical domain. Compared to participants in the lowest quartile, those in the highest quartile of street-view % total greenspace were more likely to be non-Hispanic White (86% vs. 39%) and had lower mean SES index scores (-0.65 vs. 0.25) (Table 1). The mean (SD; range) values were as follows: % total greenspace: 36.8 (12.3; 6.3–67.5), % trees: 28.4 (10.5; 2.2–59.0), % grass: 6.2 (3.5; 0.3–16.2), and % other greenspace: 2.2 (1.0; 0.1–9.5).

**Table 1:** Participant characteristics overall and by quartile of street-view total greenspace (trees + grass + other greenspace)

		% total greenspace quartile			
	Overall	Q1	Q2	Q3	Q4
	*n* = 767	*n* = 190	*n* = 184	*n* = 198	*n* = 195
	**N (%) or Mean (SD)**				
**Baseline characteristics**					
Race and ethnicity, %					
Hispanic	70 (9)	28 (15)	19 (10)	16 (8)	7 (4)
Non-Hispanic White	519 (68)	73 (39)	123 (67)	156 (79)	167 (86)
Non-Hispanic Black	113 (15)	68 (36)	27 (15)	10 (5)	8 (4)
Non-Hispanic Asian	42 (5)	8 (4)	10 (5)	13 (7)	11 (6)
> 1 race or other	22 (3)	12 (6)	5 (3)	3 (2)	2 (1)
College graduate, %	564 (74)	93 (49)	130 (71)	163 (82)	178 (91)
Married or cohabiting, %	708 (93)	159 (84)	166 (90)	192 (97)	191 (98)
Household income >$70,000/y, %	457 (64)	64 (40)	110 (63)	133 (71)	150 (80)
Neighborhood median household income,	57.1 (21.5)	43.1 (17.1)	54.0 (16.6)	63.2 (20.5)	67.6 (22.2)
$1000s/y^1^					
SES index, z-score^2^	-0.04 (0.92)	-0.65 (0.81)	-0.13 (0.87)	0.20 (0.89)	0.25 (0.86)
**Residential greenspace in 2012–2016**					
Street View-based 500 m					
% total greenspace	36.8 (12.3)	20.7 (4.4)	32.4 (2.9)	40.9 (2.2)	52.5 (5.3)
% trees	28.4 (10.5)	15.7 (4.0)	24.6 (3.2)	30.9 (3.1)	41.9 (6.2)
% grass	6.2 (3.5)	3.0 (2.6)	5.4 (2.8)	7.9 (2.7)	8.4 (3.1)
% other greenspace	2.2 (1.0)	2.0 (0.8)	2.4 (1.0)	2.2 (0.9)	2.3 (1.1)
Satellite-based NDVI 270 m	0.63 (0.13)	0.46 (0.10)	0.59 (0.08)	0.68 (0.06)	0.75 (0.05)
*Internal z-scores*					
Street View-based					
% total greenspace	0.02 (1.01)	-1.30 (0.36)	-0.34 (0.24)	0.36 (0.18)	1.31 (0.44)
% trees	0.04 (1.00)	-1.18 (0.38)	-0.33 (0.30)	0.28 (0.29)	1.33 (0.59)
% grass	-0.04 (0.96)	-0.90 (0.70)	-0.25 (0.77)	0.41 (0.73)	0.55 (0.84)
% other greenspace	0.02 (0.99)	-0.17 (0.84)	0.23 (1.01)	-0.04 (0.89)	0.05 (1.17)
Satellite-based NDVI	0.02 (1.00)	-1.25 (0.74)	-0.22 (0.64)	0.45 (0.46)	0.97 (0.39)
**Mid-life Visit in 2017–2021**					
Age, years	51.0 (5.1)	49.5 (6.5)	50.9 (4.7)	51.9 (4.2)	51.5 (4.3)
Life’s Essential 8, overall score^3^	72.3 (13.9)	69.1 (13.9)	71.6 (13.5)	73.1 (13.9)	75.3 (13.5)
Behavioral Domain^4^	72.6 (18.1)	69.5 (18.2)	71.7 (18.0)	73.2 (18.2)	75.9 (17.4)
Biomedical Domain^5^	69.0 (22.5)	65.2 (22.5)	69.1 (21.3)	69.6 (23.2)	72.0 (22.7)
*Components*					
Diet	48.1 (16.1)	46.2 (17.4)	48.5 (16.8)	48.8 (15.2)	48.7 (15.2)
Physical activity	82.5 (33.8)	78.7 (36.9)	78.8 (37.1)	82.6 (32.9)	89.8 (26.4)
Sleep	84.8 (20.9)	78.4 (25.5)	85.3 (18.7)	86.4 (20.0)	89.0 (17.1)
Avoidance of smoking	90.0 (22.1)	86.0 (27.9)	89.7 (22.5)	92.4 (17.2)	91.7 (19.0)
Body mass index	65.9 (34.1)	56.0 (35.1)	64.1 (34.2)	69.1 (33.9)	73.9 (31.1)
Blood pressure	77.3 (30.2)	69.4 (33.4)	79.3 (28.6)	81.3 (27.5)	79.0 (29.8)
Blood lipids	67.1 (31.3)	73.0 (31.7)	65.8 (31.1)	64.1 (31.8)	65.6 (30.2)
Blood glucose	90.0 (19.3)	85.4 (22.3)	90.7 (18.2)	90.0 (19.6)	94.2 (15.3)

NDVI: Normalized Difference Vegetation Index; SES: socioeconomic status

1. Neighborhood-level median household income based on participants’ residential addresses at enrollment, linked to 2000 census data

2. SES index comprising individual-level metrics (educational attainment, marital status, annual household income, and receipt of public assistance) and neighborhood-level metrics (median neighborhood income and percentage of neighborhood below the poverty line based on census tracts)

3. Possible score range 0-100, higher indicates healthier outcome

4. Behavioral domain: diet, physical activity, smoking status, sleep duration

5. Biomedical domain: BMI, blood pressure, blood lipids (non-HDL cholesterol), glycemia

**Additional File 2** shows Spearman correlations of street-view greenspace metrics and NDVI. Street-view trees were moderately correlated with grass (*r* = 0.44) and very weakly correlated with other greenspace (*r* = 0.07). NDVI was strongly correlated with street-view total greenspace (*r* = 0.85) and with trees (*r* = 0.83).

**Fig. 1:**
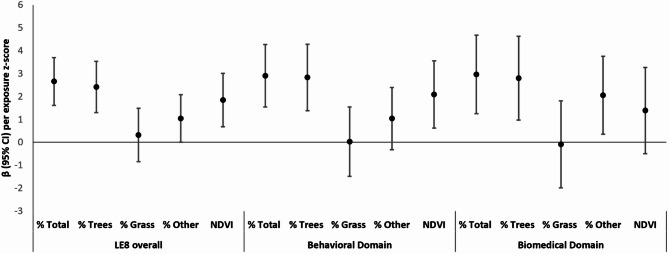
Longitudinal associations of street-view greenspace and satellite-based greenspace (NDVI) z-scores with overall CVH score and behavioral and biomedical sub-scores among midlife women in Project Viva Model 2. Adjusted for age and SES index score (incorporating both individual- and neighborhood-level metrics) and corrected for potential clustering of individuals within census tracts. We included the three greenspace components (% trees, % grass, % other greenspace) in the same model. We analyzed % total greenspace and NDVI separately, with each model excluding the three greenspace components.

In multivariable-adjusted models (Model 2), higher total % greenspace (per SD) was associated with a higher overall CVH score (β = 2.7; 95% CI: 1.6, 3.7), as well as higher behavioral (β = 2.9; 95% CI: 1.5, 4.3) and biomedical (β = 3.0; 95% CI: 1.3, 4.8) sub-scores (Table 2; Fig. 1). Further adjustment for race and ethnicity (Model 3) attenuated these associations, but they remained significant for overall (β = 1.7; 95% CI: 0.6, 2.7), behavioral (β = 2.0; 95% CI: 0.6, 3.4), and biomedical (β = 1.9; 95% CI: 0.1, 3.7) CVH scores (Table 2).

**Table 2:** Longitudinal associations of street-view greenspace and satellite-based greenspace (NDVI) z-scores with overall CVH score and behavioral and biomedical sub-scores among midlife women in project Viva

Life’s Essential 8 outcome	Greenspace exposure	Model 1	Model 2	Model 3
*(0-100 points*,* higher = better)*	(per internal z-score)	β (95% CI)		
Overall score	% Total greenspace	**2.5 (1.5**,** 3.4)**	**2.7 (1.6**,** 3.7)**	**1.7 (0.6**,** 2.7)**
	% Trees	**2.3 (1.2**,** 3.4)**	**2.4 (1.3**,** 3.5)**	**1.7 (0.5**,** 2.8)**
	% Grass	0.3 (-0.9, 1.4)	0.3 (-0.9, 1.5)	-0.1 (-1.2, 1.1)
	% Other	**1.1 (0.0**,** 2.1)**	**1.0 (0.0**,** 2.1)**	0.8 (-0.2, 1.8)
	NDVI 270 m	**1.7 (0.6**,** 2.7)**	**1.8 (0.7**,** 3.0)**	0.7 (-0.5, 1.9)
Behavioral Domain	% Total greenspace	**2.7 (1.4**,** 4.0)**	**2.9 (1.5**,** 4.3)**	**2.0 (0.6**,** 3.4)**
	% Trees	**2.7 (1.3**,** 4.1)**	**2.8 (1.4**,** 4.3)**	**2.1 (0.7**,** 3.6)**
	% Grass	0.0 (-1.5, 1.5)	0.0 (-1.5, 1.5)	-0.3 (-1.8, 1.2)
	% Other	1.2 (-0.2, 2.5)	1.0 (-0.3, 2.4)	0.8 (-0.5, 2.2)
	NDVI 270 m	**1.9 (0.6**,** 3.3)**	**2.1 (0.6**,** 3.6)**	1.0 (-0.5, 2.5)
Biomedical Domain	% Total greenspace	**2.7 (1.1**,** 4.3)**	**3.0 (1.3**,** 4.8)**	**1.9 (0.1**,** 3.7)**
	% Trees	**2.7 (0.9**,** 4.4)**	**2.8 (1.0**,** 4.7)**	**2.0 (0.1**,** 3.9)**
	% Grass	-0.2 (-2.1, 1.7)	-0.1 (-2.0, 1.9)	-0.5 (-2.5, 1.4)
	% Other	**2.1 (0.4**,** 3.8)**	**2.2 (0.4**,** 3.9)**	**1.9 (0.2**,** 3.6)**
	NDVI 270 m	1.2 (-0.5, 2.9)	1.4 (-0.5, 3.3)	0.1 (-1.9, 2.0)

NDVI: Normalized Difference Vegetation Index; SES: socioeconomic status

Model 1. Unadjusted

Model 2. Adjusted for age and SES index score (incorporating both individual- and neighborhood-level metrics)

Model 3. Model 2 + race and ethnicity

All models corrected for potential clustering of individuals within census tracts

We included the three greenspace components (% trees, % grass, % other greenspace) in the same model. We analyzed % total greenspace and NDVI separately, with each model excluding the three greenspace components

Bold font indicates results that are statistically significant (95% CI excludes the null)

When analyzing the individual components of greenspace within the same model, % trees was associated with overall (β = 2.4; 95% CI: 1.3, 3.5), behavioral (β = 2.8; 95% CI: 1.4, 4.3), and biomedical (β = 2.8; 95% CI: 1.0, 4.7) CVH scores. Additionally, % other greenspace was associated with biomedical CVH scores (β = 2.2; 95% CI: 0.4, 3.9), whereas associations for % grass were non-significant (Table 2; Fig. 1). In models where NDVI was the sole exposure, NDVI (per SD) was associated with overall (β = 1.8; 95% CI: 0.7, 3.0) and behavioral (β = 2.1; 95% CI: 0.6, 3.6), but not biomedical (β = 1.4; 95% CI: -0.5, 3.3) CVH scores (Table 2; Fig. 1).

In a sensitivity analysis, we examined street-view exposures with additional adjustment for satellite-based NDVI, and the associations strengthened. In these models, higher total % greenspace (per SD) was associated with a higher overall CVH score (β = 3.9; 95% CI: 2.0, 5.8), as well as higher behavioral (β = 4.0; 95% CI: 1.5, 6.6) and biomedical (β = 5.3; 95% CI: 2.2, 8.3) sub-scores. In models including the three greenspace components, with additional adjustment for NDVI, higher % tree cover was associated with higher overall (β = 3.3; 95% CI: 1.6, 5.0), behavioral (β = 3.6; 95% CI: 1.3, 5.9), and biomedical (β = 4.4; 95% CI: 1.6, 7.2) CVH scores (**Additional File 3**,** Model 2**).

**Fig. 2:**
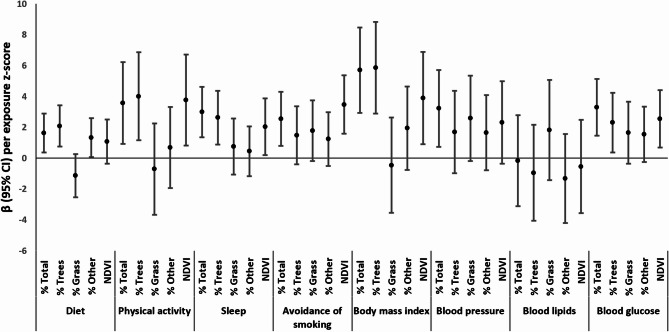
Longitudinal associations of street-view greenspace and satellite-based greenspace (NDVI) z-scores with LE8 component scores among midlife women in Project Viva Model 2. Adjusted for age and SES index score (incorporating both individual- and neighborhood-level metrics) and corrected for potential clustering of individuals within census tracts We included the three greenspace components (% trees, % grass, % other greenspace) in the same model. We analyzed % total greenspace and NDVI separately, with each model excluding the three greenspace components

When examining individual CVH components, higher total % greenspace (per SD) was associated with higher scores for each CVH component, except lipids: diet (β = 1.6; 95% CI: 0.3, 2.9), physical activity (β = 3.6; 95% CI: 0.9, 6.2), sleep (β = 3.0; 95% CI: 1.4, 4.6), smoking avoidance (β = 2.5; 95% CI: 0.7, 4.4), BMI (β = 5.8; 95% CI: 3.0, 8.6), blood pressure (β = 3.3; 95% CI: 0.7, 5.8) and blood glucose (β = 3.3; 95% CI: 1.4, 5.2) (Table 3; Fig. 2, **Model 2**). Higher % trees was also associated with higher scores for most CVH components, including diet (β = 2.1; 95% CI: 0.7, 3.4), physical activity (β = 4.0; 95% CI: 1.2, 6.9), sleep (β = 2.6; 95% CI: 0.9, 4.4), BMI (β = 5.8; 95% CI: 2.8, 8.8), and blood glucose (β = 2.2; 95% CI: 0.3, 4.2) (Table 3; Fig. 2, **Model 2**).

**Table 3:** Longitudinal associations of street-view greenspace and satellite-based greenspace (NDVI) z-scores with LE8 component scores among midlife women in project Viva

Life’s Essential 8 outcome	Greenspace exposure	Model 1	Model 2	Model 3
*(0-100 points*,* higher = better)*	(per internal z-score)	β (95% CI)		
Diet	% Total greenspace	**1.2 (0.0**,** 2.4)**	**1.6 (0.3**,** 2.9)**	**1.4 (0.1**,** 2.8)**
	% Trees	**1.8 (0.4**,** 3.1)**	**2.1 (0.7**,** 3.4)**	**2.0 (0.6**,** 3.4)**
	% Grass	-1.3 (-2.7, 0.2)	-1.2 (-2.6, 0.2)	-1.2 (-2.6, 0.2)
	% Other	**1.4 (0.1**,** 2.7)**	**1.3 (0.0**,** 2.5)**	1.2 (-0.1, 2.5)
	NDVI 270 m	0.6 (-0.7, 2.0)	1.1 (-0.4, 2.5)	0.8 (-0.7, 2.4)
Physical activity	% Total greenspace	**4.1 (1.7**,** 6.6)**	**3.6 (0.9**,** 6.2)**	2.1 (-0.7, 4.8)
	% Trees	**4.5 (1.8**,** 7.2)**	**4.0 (1.2**,** 6.9)**	2.8 (-0.1, 5.7)
	% Grass	-0.4 (-3.4, 2.5)	-0.7 (-3.7, 2.3)	-1.2 (-4.2, 1.7)
	% Other	0.8 (-1.9, 3.4)	0.7 (-1.9, 3.3)	0.2 (-2.4, 2.8)
	NDVI 270 m	**4.4 (1.8**,** 7.0)**	**3.8 (0.8**,** 6.7)**	2.2 (-0.8, 5.2)
Sleep	% Total greenspace	**3.4 (1.9**,** 5.0)**	**3.0 (1.4**,** 4.6)**	**1.7 (0.1**,** 3.4)**
	% Trees	**3.0 (1.3**,** 4.7)**	**2.6 (0.9**,** 4.4)**	1.6 (-0.1, 3.3)
	% Grass	1.0 (-0.8, 2.8)	0.8 (-1.1, 2.6)	0.3 (-1.5, 2.1)
	% Other	0.6 (-1.0, 2.2)	0.4 (-1.2, 2.1)	0.1 (-1.5, 1.7)
	NDVI 270 m	**2.7 (1.0**,** 4.3)**	**2.0 (0.2**,** 3.9)**	0.6 (-1.2, 2.5)
Avoidance of smoking	% Total greenspace	**2.5 (0.7**,** 4.2)**	**2.5 (0.7**,** 4.4)**	**2.2 (0.3**,** 4.1)**
	% Trees	1.5 (-0.4, 3.4)	1.6 (-0.4, 3.5)	1.3 (-0.6, 3.3)
	% Grass	1.5 (-0.5, 3.6)	1.6 (-0.4, 3.6)	1.4 (-0.6, 3.4)
	% Other	1.4 (-0.4, 3.2)	1.2 (-0.6, 3.0)	1.2 (-0.6, 3.0)
	NDVI 270 m	**3.2 (1.4**,** 4.9)**	**3.5 (1.6**,** 5.4)**	**3.2 (1.2**,** 5.2)**
Body mass index	% Total greenspace	**6.6 (4.0**,** 9.2)**	**5.8 (3.0**,** 8.6)**	**3.3 (0.4**,** 6.1)**
	% Trees	**6.6 (3.7**,** 9.5)**	**5.8 (2.8**,** 8.8)**	**4.0 (1.0**,** 6.9)**
	% Grass	0.0 (-3.1, 3.1)	-0.4 (-3.5, 2.8)	-1.5 (-4.6, 1.5)
	% Other	2.2 (-0.6, 4.9)	2.1 (-0.6, 4.8)	1.6 (-1.1, 4.2)
	NDVI 270 m	**5.2 (2.4**,** 7.9)**	**3.8 (0.8**,** 6.9)**	1.2 (-1.8, 4.2)
Blood pressure	% Total greenspace	**3.4 (1.0**,** 5.7)**	**3.3 (0.7**,** 5.8)**	1.4 (-1.2, 4.1)
	% Trees	1.9 (-0.7, 4.5)	1.9 (-0.9, 4.6)	0.4 (-2.3, 3.2)
	% Grass	2.4 (-0.4, 5.2)	2.3 (-0.5, 5.1)	1.6 (-1.1, 4.4)
	% Other	1.6 (-0.9, 4.1)	1.8 (-0.7, 4.2)	1.2 (-1.2, 3.7)
	NDVI 270 m	2.3 (-0.1, 4.8)	2.3 (-0.4, 5.0)	0.3 (-2.4, 3.1)
Blood lipids	% Total greenspace	-2.3 (-5.1, 0.5)	-0.2 (-3.2, 2.8)	-0.2 (-3.3, 3.0)
	% Trees	-2.6 (-5.7, 0.5)	-0.9 (-4.1, 2.3)	-0.9 (-4.1, 2.4)
	% Grass	0.6 (-2.8, 3.9)	1.6 (-1.7, 4.9)	1.7 (-1.7, 5.1)
	% Other	-0.8 (-3.8, 2.2)	-0.8 (-3.7, 2.1)	-0.7 (-3.6, 2.2)
	NDVI 270 m	**-2.9 (-5.7**,** 0.0)**	-0.6 (-3.6, 2.5)	-0.6 (-3.8, 2.6)
Blood glucose	% Total greenspace	**2.9 (1.3**,** 4.6)**	**3.3 (1.4**,** 5.2)**	**2.3 (0.4**,** 4.2)**
	% Trees	**2.0 (0.1**,** 3.8)**	**2.2 (0.3**,** 4.2)**	1.6 (-0.3, 3.6)
	% Grass	1.6 (-0.4, 3.6)	1.7 (-0.3, 3.8)	1.1 (-0.9, 3.1)
	% Other	1.6 (-0.2, 3.4)	1.6 (-0.2, 3.4)	1.3 (-0.5, 3.1)
	NDVI 270 m	**2.2 (0.6**,** 3.9)**	**2.5 (0.6**,** 4.4)**	1.5 (-0.4, 3.5)

NDVI: Normalized Difference Vegetation Index; SES: socioeconomic status

Model 1. Unadjusted

Model 2. Adjusted for age and SES index score (incorporating both individual- and neighborhood-level metrics)

Model 3. Model 2 + race and ethnicity

All models corrected for potential clustering of individuals within census tracts

We included the three greenspace components (% trees, % grass, % other greenspace) in the same model. We analyzed % total greenspace and NDVI separately, with each model excluding the three greenspace components

Bold font indicates results that are statistically significant (95% CI excludes the null)

## Discussion

Our findings suggest that greater exposure to visible greenspace, particularly trees, is prospectively associated with better subsequent cardiovascular health among women in midlife, even after adjusting for individual- and neighborhood-level socioeconomic status. Specifically, we observed significant positive associations of overall greenspace with Life’s Essential 8 CVH scores, as well as its behavioral and biomedical sub-scores about 5 years later. These results contribute to the growing body of evidence linking neighborhood environmental factors to cardiovascular health and highlight the potential role of greenspace exposure in promoting heart health during midlife [8, 9, 10, 11, 12, 13, 41, 42, 43].

A key finding of our study is the differential associations of street-view greenspace components with CVH. While total greenspace was positively associated with CVH scores, trees demonstrated the strongest associations across both behavioral and biomedical health domains. This finding suggests that presence of trees in the neighborhood, rather than other forms of greenspace such as grass, may play a particularly important role in influencing health outcomes. The observed associations between the proportion of trees and individual CVH components, including diet, physical activity, sleep, BMI, and blood glucose, further support this hypothesis. Trees may promote physical activity by providing shade, encouraging outdoor exercise, and enhancing neighborhood walkability [18]. Additionally, increased tree coverage has been linked to reduced air pollution and heat exposure, both of which may contribute to improved metabolic and cardiovascular health [17].

Our study also found that the associations between greenspace and CVH were attenuated but remained significant after adjusting for race and ethnicity. This suggests that racial and ethnic disparities in greenspace access may partially explain differences in CVH, highlighting the importance of urban planning efforts that support tree planting and maintenance in historically under-resourced communities [44].

A recent systematic review and meta-analysis of 53 studies, encompassing over 100 million individuals from 18 countries, identified a positive association between greenspace exposure and CVH [43]. Specifically, the study reported that a 0.1-unit increase in NDVI was associated with 2–3% lower odds of cardiovascular disease mortality (OR = 0.97, 95% CI: 0.96, 0.99), ischemic heart disease mortality (OR = 0.98, 95% CI: 0.96, 1.00), cerebrovascular disease mortality (OR = 0.98, 95% CI: 0.97, 1.00), and stroke incidence/prevalence (OR = 0.98, 95% CI: 0.96, 0.99). However, as this meta-analysis relied on satellite-based NDVI, it could not differentiate between various types of greenspaces. In contrast, our study utilized street-view greenspace metrics, allowing us to examine specific greenspace components perceived at eye level. Additionally, our study used a CVH score rather than mortality outcomes, offering a more comprehensive assessment of cardiovascular health.

Notably, among offspring of women in this same cohort (Project Viva), [45] Yi et al. did not find a longitudinal association between street-view greenspace and LE8 CVH scores [21]. However, Yi et al. observed a cross-sectional association of street-view trees with CVH scores in late adolescence, but not in mid-childhood or early adolescence. This discrepancy may be attributed to differences in how participants utilized greenspace across life stages. This difference might be because older adolescents had greater independence to explore and engage with various types of greenspace in their neighborhoods compared to when they were younger [46].

The strengths of our study include the use of deep learning algorithms to quantify specific types of greenspace from street-view images, a novel and complementary approach to traditional satellite-based measures that has increased policy relevance. Additionally, our prospective design with a well-characterized cohort allowed us to assess associations between greenspace and CVH over time. However, several limitations should be considered. First, as an observational study, we cannot draw causal inferences. We linked greenspace metrics to residential addresses, with no information on participants’ time-activity patterns or whether they actually spent time in greenspaces around their homes. While our study population was socioeconomically diverse, the majority of participants were non-Hispanic White and college-educated, which may limit generalizability to other populations.

Additionally, we recruited participants from primarily urban and suburban areas of Eastern Massachusetts, and although the cohort is now nationally distributed, many individuals have remained in the region. This could restrict generalizability if, for example, the composition of tree species differs from other regions. Our findings might not fully capture the experiences of individuals living in rural areas, or more western or southern regions, where greenspace types and access may differ from the Northeast. Furthermore, differences between participants included and excluded from the study might have led to retention bias toward individuals of higher socioeconomic status. This may have influenced observed associations, as higher SES individuals may be more likely to reside in greener neighborhoods and have greater access to resources that support CVH. Our analysis controlled for this issue to a certain degree by adjusting for sociodemographic factors related to inclusion in this analysis. Finally, estimates of street-view greenspace based on Google Street View images have some limitations. Vegetation may be blocked by buildings, vehicles, or other objects, and differences in camera angle or distance can affect how much greenery is captured. Vegetation farther from the camera may be less visible, which could lead to underestimating greenspace in areas with larger setbacks. These factors could introduce nondifferential measurement error, which may attenuate observed associations of greenspace exposure with cardiovascular health outcomes.

Future research should explore potential mechanisms underlying the observed associations, including the roles of psychosocial stress reduction, improved air quality, and increased opportunities for physical activity. Additionally, studies incorporating longitudinal assessments of greenspace exposure and interventions aimed at increasing tree cover could provide further insights into causal relationships. Given the public health implications, urban planning efforts that enhance tree canopy coverage in residential neighborhoods may serve as an effective strategy for improving cardiovascular health, particularly among midlife women.

### Perspectives and significance

Our study underscores the broader public health implications of greenspace exposure, particularly trees, in improving cardiovascular health among midlife women. As urbanization continues to expand, integrating greenspace into city planning could offer a sustainable approach to mitigating cardiovascular disease risk. Future research should investigate the interplay between greenspace, social determinants of health, and policy interventions to ensure equitable access to green environments. By prioritizing greenspace expansion and nature-based solutions, policymakers and urban planners may enhance both environmental sustainability and population health outcomes.

## Electronic supplementary material

Below is the link to the electronic supplementary material.

Supplementary Material 1

## Data Availability

Data included in this manuscript are not publicly available because Project Viva’s historic consents did not allow for public data sharing. In accordance with Project Viva policies, datasets are available upon reasonable request via the Project Viva ROADMaP portal at: https://vivaroadmap.net/users/sign_up? invitation_code=welcome-to-viva-roadmap. All data collection instruments are also available via the ROADMaP.

## References

[1] Office of Disease Prevention and Health Promotion. (n.d.). *Improve cardiovascular health in adults* — *HDS-01*. Healthy People 2030. U.S. Department of Health and Human Services. Retrieved 2/22/25, from https://odphp.health.gov/healthypeople/objectives-and-data/browse-objectives/heart-disease-and-stroke/improve-cardiovascular-health-adults-hds-01

[2] Lloyd-Jones DM, Allen NB, Anderson CAM, Black T, Brewer LC, Foraker RE, American Heart Association. Life’s Essential 8: Updating and Enhancing the American Heart Association’s Construct of Cardiovascular Health: A Presidential Advisory From the American Heart Association. Circulation. 2022;146(5):e18-e43. doi: 10.1161/CIR.0000000000001078. Epub 2022 Jun 29. PMID: 35766027; PMCID: PMC10503546. 10.1161/CIR.0000000000001078 PMC10503546 35766027

[3] El Khoudary SR, Aggarwal B, Beckie TM, Hodis HN, Johnson AE, Langer RD, Limacher MC, Manson JE, Stefanick ML, Allison MA, American Heart Association Prevention Science Committee of the Council on Epidemiology and Prevention; and Council on Cardiovascular and Stroke Nursing. Menopause transition and cardiovascular disease risk: implications for timing of early prevention: A scientific statement from the American heart association. Circulation. 2020;142(25):e506–32. 10.1161/CIR.0000000000000912. Epub 2020 Nov 30. PMID: 33251828. 33251828 10.1161/CIR.0000000000000912

[4] Zheng Y, Manson JE, Yuan C, Liang MH, Grodstein F, Stampfer MJ, Willett WC, Hu FB. Associations of weight gain from early to middle adulthood with major health outcomes later in life. JAMA. 2017;318(3):255–69. 10.1001/jama.2017.7092. PMID: 28719691; PMCID: PMC5817436. 28719691 10.1001/jama.2017.7092 PMC5817436

[5] Maas AH, Franke HR. Women’s health in menopause with a focus on hypertension. Neth Heart J. 2009;17(2):68–72. 10.1007/BF03086220. PMID: 19247469; PMCID: PMC2644382. 19247469 10.1007/BF03086220 PMC2644382

[6] Woodard GA, Brooks MM, Barinas-Mitchell E, Mackey RH, Matthews KA, Sutton-Tyrrell K. Lipids, menopause, and early atherosclerosis in study of women’s health across the Nation heart women. Menopause. 2011;18(4):376–84. 10.1097/gme.0b013e3181f6480e. PMID: 21107300; PMCID: PMC3123389. 21107300 10.1097/gme.0b013e3181f6480e PMC3123389

[7] Ma H, Wang X, Xue Q, Li X, Liang Z, Heianza Y, et al. Cardiovascular health and life expectancy among adults in the united States. Circulation. 2023;147(15):1137–46. 10.1161/CIRCULATIONAHA.122.062457. Epub 2023 Apr 10. PMID: 37036905; PMCID: PMC10165723. 37036905 10.1161/CIRCULATIONAHA.122.062457 PMC10165723

[8] Bodicoat DH, O’Donovan G, Dalton AM, Gray LJ, Yates T, Edwardson C, et al. The association between neighbourhood greenspace and type 2 diabetes in a large cross-sectional study. BMJ Open. 2014;4(12):e006076. 10.1136/bmjopen-2014-006076. PMID: 25537783; PMCID: PMC4275673. 25537783 10.1136/bmjopen-2014-006076 PMC4275673

[9] Raaschou-Nielsen O, Poulsen AH, Ketzel M, Frohn LM, Roswall N, Hvidtfeldt UA, et al. Cardiometabolic health effects of air pollution, noise, green space, and socioeconomic status: the HERMES study. Res Rep Health Eff Inst. 2024;222:1–62. PMID: 39916362. PMC11816022 39916362

[10] Pereira G, Foster S, Martin K, Christian H, Boruff BJ, Knuiman M, et al. The association between neighborhood greenness and cardiovascular disease: an observational study. BMC Public Health. 2012;12:466. 10.1186/1471-2458-12-466. PMID: 22720780; PMCID: PMC3476430. 22720780 10.1186/1471-2458-12-466 PMC3476430

[11] Chen Z, Salerno PRVO, Dazard JE, Makhlouf MH, Deo S, Rajagopalan S, Al-Kindi S. Deep Learning Analysis of Google Street View to Assess Residential Built Environment and Cardiovascular Risk in a U.S. Midwestern Retrospective Cohort. Eur J Prev Cardio. 2025 Feb 4:zwaf038. 10.1093/eurjpc/zwaf038. Epub ahead of print. PMID: 39903569. 10.1093/eurjpc/zwaf038 PMC12319116 39903569

[12] Alexeeff SE, Roy A, Shan J, Liu X, Messier K, Apte JS, et al. High-resolution mapping of traffic related air pollution with Google street view cars and incidence of cardiovascular events within neighborhoods in Oakland, CA. Environ Health. 2018;17(1):38. 10.1186/s12940-018-0382-1. PMID: 29759065; PMCID: PMC5952592. 29759065 10.1186/s12940-018-0382-1 PMC5952592

[13] Yeager R, Keith RJ, Riggs DW, Fleischer D, Browning MHEM, Ossola A, et al. Intra-neighborhood associations between residential greenness and blood pressure. Sci Total Environ. 2024;946:173788. 10.1016/j.scitotenv.2024.173788. Epub 2024 Jun 18. PMID: 38901580. 38901580 10.1016/j.scitotenv.2024.173788 PMC12061022

[14] Jennings V, Bamkole O. The relationship between social cohesion and urban green space: an avenue for health promotion. Int J Environ Res Public Health. 2019;16(3):452. 10.3390/ijerph16030452. PMID: 30720732; PMCID: PMC6388234. 30720732 10.3390/ijerph16030452 PMC6388234

[15] Kanning M, Yi L, Yang CH, Niermann C, Fina S. Mental health in urban environments: Uncovering the black box of Person-Place interactions requires interdisciplinary approaches. JMIR Mhealth Uhealth. 2023;11:e41345. 10.2196/41345. PMID: 37166963; PMCID: PMC10214119. 37166963 10.2196/41345 PMC10214119

[16] Beele E, Aerts R, Reyniers M, Somers B. Urban green space, human heat perception and sleep quality: A repeated cross-sectional study. Environ Res Doi: 10.1016/j.envres.2024.120129. Epub 2024 Oct 9. PMID: 39389201. 10.1016/j.envres.2024.120129 39389201

[17] Bloemsma LD, Gehring U, Klompmaker JO, Hoek G, Janssen NAH, Lebret E et al. Green space, air pollution, traffic noise and cardiometabolic health in adolescents: The PIAMA birth cohort. Environ Int. 2019;131:104991. 10.1016/j.envint.2019.104991. Epub 2019 Jul 11. PMID: 31302482. 10.1016/j.envint.2019.104991 31302482

[18] Lu Y. Using Google street view to investigate the association between street greenery and physical activity. Landsc Urban Plann. 2019;191:103435. 10.1016/j.landurbplan.2018.08.029.

[19] Larkin A, Hystad P. Evaluating street view exposure measures of visible green space for health research. J Expo Sci Environ Epidemiol. 2019;29(4):447–56. 10.1038/s41370-018-0017-1. Epub 2018 Jan 19. PMID: 29352209. 29352209 10.1038/s41370-018-0017-1

[20] Browning MHEM, Locke DH, Konijnendijk C, Labib SM, Rigolon A, Yeager R, et al. Measuring the 3-30-300 rule to help cities Meet nature access thresholds. Sci Total Environ. 2024;907:167739. 10.1016/j.scitotenv.2023.167739. Epub 2023 Oct 11. PMID: 37832672; PMCID: PMC11090249. 37832672 10.1016/j.scitotenv.2023.167739 PMC11090249

[21] Yi L, Rifas-Shiman S, Pescador Jimenez M, Lin PD, Suel E, Hystad P, et al. Assessing greenspace and cardiovascular health through deep-learning analysis of street-view imagery in a cohort of US children. Environ Res. 2025;265:120459. 10.1016/j.envres.2024.120459. Epub 2024 Nov 26. PMID: 39603586; PMCID: PMC11742899. 39603586 10.1016/j.envres.2024.120459 PMC11742899

[22] Yi L, Hart JE, Roscoe C, Mehta UV, Pescador Jimenez M, Lin PD, Suel E, Hystad P, Hankey S, Zhang W, Okereke OI, Laden F, James P. Greenspace and depression incidence in the US-based nationwide nurses’ health study II: A deep learning analysis of street-view imagery. Environ Int. 2025;198:109429. 10.1016/j.envint.2025.109429. Epub ahead of print. PMID: 40209395. 40209395 10.1016/j.envint.2025.109429 PMC12224280

[23] Jimenez MP, Suel E, Rifas-Shiman SL, Hystad P, Larkin A, Hankey S, et al. Street-view greenspace exposure and objective sleep characteristics among children. Environ Res. 2022;214(Pt 1):113744. 10.1016/j.envres.2022.113744. Epub 2022 Jun 25. PMID: 35760115; PMCID: PMC9930007. 35760115 10.1016/j.envres.2022.113744 PMC9930007

[24] Yi L, Harnois-Leblanc S, Rifas-Shiman SL, Suel E, Pescador Jimenez M, et al. Satellite-Based and Street-View green space and adiposity in US children. JAMA Netw Open. 2024;7(12):e2449113. 10.1001/jamanetworkopen.2024.49113. PMID: 39636637; PMCID: PMC11621986. 39636637 10.1001/jamanetworkopen.2024.49113 PMC11621986

[25] Lu Y. The association of urban greenness and walking behavior: using Google street view and deep learning techniques to estimate residents’ exposure to urban greenness. Int J Environ Res Public Health. 2018;15(8):1576. 10.3390/ijerph15081576. PMID: 30044417; PMCID: PMC6121356. 30044417 10.3390/ijerph15081576 PMC6121356

[26] Xiao X, Wang R, Knibbs LD, Jalaludin B, Heinrich J, Markevych I, et al. Street view greenness is associated with lower risk of obesity in adults: findings from the 33 Chinese community health study. Environ Res. 2021;200:111434. 10.1016/j.envres.2021.111434. Epub 2021 Jun 2. PMID: 34087194. 34087194 10.1016/j.envres.2021.111434

[27] Rifas-Shiman SL, Aris IM, Switkowski KM, Young J, Fleisch AF, James-Todd T, et al. Cohort profile update: project Viva mothers. Int J Epidemiol. 2023;52(6):e332–9. 10.1093/ije/dyad137. PMID: 37875013; PMCID: PMC10749767. 37875013 10.1093/ije/dyad137 PMC10749767

[28] Oken E, Baccarelli AA, Gold DR, Kleinman KP, Litonjua AA, De Meo D, et al. Cohort profile: project Viva. Int J Epidemiol. 2015;44(1):37–48. 10.1093/ije/dyu008. Epub 2014 Mar 16. PMID: 24639442; PMCID: PMC4339753. 24639442 10.1093/ije/dyu008 PMC4339753

[29] Zhao H, Shi J, Qi X, Wang X, Jia J, Recognition P. (CVPR). IEEE, pp. 6230–6239. 10.1109/CVPR.2017.660.

[30] Zhou B, Zhao H, Puig X, et al. Int J Comput Vis. 2019;127(3):302–21. 10.1007/s11263-018-1140-0. Semantic understanding of scenes through the ADE20K dataset.

[31] Luo YN, Huang WZ, Liu XX, Markevych I, Bloom MS, Zhao T, et al. Greenspace with overweight and obesity: A systematic review and meta-analysis of epidemiological studies up to 2020. Obes Rev. 2020;21(11):e13078. 10.1111/obr.13078. Epub 2020 Jul 16. PMID: 32677149. 32677149 10.1111/obr.13078

[32] James P, Suel E, Lin P-I, Debby, Hart J, Rimm EB, Laden. Francine and Hystad, Perry and Hankey, Steve and Larkin, Andrew and Zhang, Wenwen and Klompmaker, Jochem O. and Coull, Brent and Yi, Li and Jimenez, Marcia Pescador, Assessing Greenspace and Cardiovascular Disease Risk Through Deep Learning Analysis of Street-View Imagery in the Us-Based Nationwide Nurses’ Health Study. Available at SSRN: https://ssrn.com/abstract=4943757 or 10.2139/ssrn.4943757 10.1097/EE9.0000000000000442 PMC12818865 41567830

[33] Kwan MP. The uncertain geographic context problem. Ann Assoc Am Geogr. 2012;102(5):958–68. 10.1080/00045608.2012.687349.

[34] Wilt GE, Roscoe CJ, Hu CR, Mehta UV, Coull BA, Hart JE, Gortmaker S, Laden F, James P. Minute level smartphone derived exposure to greenness and consumer wearable derived physical activity in a cohort of US women. Environ Res. 2023;237(Pt 2):116864. 10.1016/j.envres.2023.116864. Epub 2023 Aug 28. PMID: 37648192; PMCID: PMC11146007. 37648192 10.1016/j.envres.2023.116864 PMC11146007

[35] Nichols AR, Rifas-Shiman SL, Switkowski KM, Zhang M, Young JG, Hivert MF, et al. History of infertility and midlife cardiovascular health in female individuals. JAMA Netw Open. 2024;7(1):e2350424. 10.1001/jamanetworkopen.2023.50424. PMID: 38180761; PMCID: PMC10770770. 38180761 10.1001/jamanetworkopen.2023.50424 PMC10770770

[36] Zheng Y, Huang T, Guasch-Ferre M, Hart J, Laden F, Chavarro J, et al. Estimation of life’s essential 8 score with incomplete data of individual metrics. Front Cardiovasc Med. 2023;10:1216693. 10.3389/fcvm.2023.1216693. PMID: 37564908; PMCID: PMC10410141. 37564908 10.3389/fcvm.2023.1216693 PMC10410141

[37] Flanagin A, Frey T, Christiansen SL, AMA Manual of Style Committee. Updated Guidance on the Reporting of Race and Ethnicity in Medical and Science Journals. JAMA. 2021;326(7):621–627. 10.1001/jama.2021.13304. PMID: 34402850. 10.1001/jama.2021.13304 34402850

[38] Howe CJ, Bailey ZD, Raifman JR, Jackson JW. Recommendations for using causal diagrams to study Racial health disparities. Am J Epidemiol. 2022;191(12):1981–9. 10.1093/aje/kwac140. PMID: 35916384; PMCID: PMC10144617. 35916384 10.1093/aje/kwac140 PMC10144617

[39] Laubach ZM, Perng W, Cardenas A, Rifas-Shiman SL, Oken E, DeMeo D, et al. Socioeconomic status and DNA methylation from birth through mid-childhood: a prospective study in project Viva. Epigenomics. 2019;11(12):1413–27. 10.2217/epi-2019-0040. Epub 2019 Sep 11. PMID: 31509016; PMCID: PMC6802709. 31509016 10.2217/epi-2019-0040 PMC6802709

[40] Aris IM, Lin PD, Wu AJ, et al. Birth outcomes in relation to neighborhood food access and individual food insecurity during pregnancy in the environmental influences on child health outcomes (ECHO)-wide cohort study. Am J Clin Nutr. 2024;119(5):1216–26. Epub 2024 Mar 1. PMID: 38431121; PMCID: PMC11130689. 38431121 10.1016/j.ajcnut.2024.02.022 PMC11130689

[41] James P, Hart JE, Banay RF, Laden F. Exposure to greenness and mortality in a nationwide prospective cohort study of women. Environ Health Perspect. 2016;124(9):1344–52. 10.1289/ehp.1510363. Epub 2016 Apr 14. PMID: 27074702; PMCID: PMC5010419. 27074702 10.1289/ehp.1510363 PMC5010419

[42] Rojas-Rueda D, Nieuwenhuijsen MJ, Gascon M, Perez-Leon D, Mudu P. Green spaces and mortality: a systematic review and meta-analysis of cohort studies. Lancet Planet Health. 2019;3(11):e469–77. 10.1016/S2542-5196(19)30215-3. 31777338 10.1016/S2542-5196(19)30215-3 PMC6873641

[43] Liu XX, Ma XL, Huang WZ, Luo YN, He CJ, Zhong XM, et al. Green space and cardiovascular disease: A systematic review with meta-analysis. Environ Pollut. 2022;301:118990. 10.1016/j.envpol.2022.118990. Epub 2022 Feb 15. PMID: 35181451. 35181451 10.1016/j.envpol.2022.118990

[44] Mitchell R, Popham F. Effect of exposure to natural environment on health inequalities: an observational population study. Lancet. 2008;372(9650):1655-60. 10.1016/S0140-6736(08)61689-X. PMID: 18994663. 10.1016/S0140-6736(08)61689-X 18994663

[45] Rifas-Shiman SL, Aris IM, Switkowski KM, Young J, Fleisch AF, Perng W, et al. Cohort profile update: project Viva offspring. Int J Epidemiol. 2024;53(6):dyae162. 10.1093/ije/dyae162. PMID: 39657066; PMCID: PMC11630542. 39657066 10.1093/ije/dyae162 PMC11630542

[46] Prince SA, Butler GP, Rao DP, Thompson W. Evidence synthesis - Where are children and adults physically active and sedentary? - a rapid review of location-based studies. Health Promot Chronic Dis Prev Can. 2019;39(3):67–103. 10.24095/hpcdp.39.3.01. PMID: 30869472; PMCID: PMC6478053. 30869472 10.24095/hpcdp.39.3.01 PMC6478053

