# Supplementary material for: Associations of street-view greenspace exposure with cardiovascular health (Life’s Essential 8) among women in midlife
Source: Biol Sex Differ. 2025 Jul 1;16:45. doi: 10.1186/s13293-025-00718-3 (PMC12219997; doi:10.1186/s13293-025-00718-3)
Supplement: Supplementary file 1 — Supplementary Material 1 [file 13293_2025_718_MOESM1_ESM.docx]

**Additional File 1.** Baseline characteristics (at study enrollment in pregnancy) of participants in Project Viva who were included in the analytic sample v. who were excluded

| Characteristics | Overall | Included | Excluded |
| --- | --- | --- | --- |
| at enrollment | n=2100 | n=767 | n=1333 |
|  | **% or mean (SD)** | | |
| Age at enrollment, years | 31.8 (5.2) | 32.6 (5.0) | 31.3 (5.3) |
| Pre-pregnancy BMI, kg/m^2^ | 24.9 (5.5) | 24.6 (5.2) | 25.0 (5.7) |
| Neighborhood median household income, $1000s/y^1^ | 56.4 (21.3) | 57.1 (21.5) | 56.0 (21.2) |
| SES index, z-score^2^ | -0.00 (0.93) | -0.04 (0.92) | 0.02 (0.93) |
| Race and ethnicity, % |  |  |  |
| Hispanic | 9% | 9% | 9% |
| NH White | 66% | 68% | 65% |
| NH Black | 17% | 15% | 18% |
| NH Asian | 6% | 5% | 6% |
| > 1 race or other | 3% | 3% | 2% |
| College graduate, % | 65% | 74% | 59% |
| Married or cohabiting, % | 91% | 93% | 91% |
| Prenatal smoking, % |  |  |  |
| Never | 69% | 71% | 67% |
| Former | 19% | 20% | 18% |
| Smoked during pregnancy | 13% | 9% | 15% |
| Nulliparous, % | 48% | 48% | 49% |
| Household income >$70,000/y, % | 61% | 64% | 59% |

SES: socioeconomic status

1. Neighborhood-level median household income based on participants' residential addresses at enrollment, linked to 2000 census data
2. SES index comprising individual-level metrics (educational attainment, marital status, annual household income, and receipt of public assistance) and neighborhood-level metrics (median neighborhood income and percentage of neighborhood below the poverty line based on census tracts)

**Additional File 2.** Spearman correlation coefficients between street-view greenspace metrics (500 meters) and NDVI (270 meters) in 2012 – 2016, and SES index z-score^1^

|  |  | **Street view greenspace** | | | |  |
| --- | --- | --- | --- | --- | --- | --- |
|  |  | **% total greenspace** | **% trees** | **% grass** | **% other greenspace** | **NDVI** |
| % trees | r | 0.97 |  |  |  |  |
|  | p-value | <.0001 |  |  |  |  |
| % grass | r | 0.61 | 0.44 |  |  |  |
|  | p-value | <.0001 | <.0001 |  |  |  |
| % other greenspace | r | 0.04 | 0.07 | -0.27 |  |  |
|  | p-value | 0.29 | 0.04 | <.0001 |  |  |
| **NDVI** | r | 0.85 | 0.83 | 0.58 | -0.11 |  |
|  | p-value | <.0001 | <.0001 | <.0001 | 0.00 |  |
| SES index, z-score^1^ | r | 0.39 | 0.38 | 0.29 | -0.08 | 0.42 |
|  | p-value | <.0001 | <.0001 | <.0001 | 0.02 | <.0001 |

NDVI: Normalized Difference Vegetation Index; SES: socioeconomic status

1. SES index comprising individual-level metrics (educational attainment, marital status, annual household income, and receipt of public assistance) and neighborhood-level metrics (median neighborhood income and percentage of neighborhood below the poverty line based on census tracts)

**Additional File 3**. Longitudinal associations of street-view greenspace with overall CVH score and behavioral and biomedical sub-scores, adjusted for satellite-based greenspace (NDVI)

| **Life's Essential 8 outcome** | **Greenspace exposure** | **Model 1** | **Model 2** | **Model 3** |
| --- | --- | --- | --- | --- |
| *(0-100 points, higher=better)* | (per internal z-score) | β (95% CI) | | |
|  |  |  |  |  |
| Overall score | % Total greenspace | **3.6 (1.8, 5.4)** | **3.9 (2.0, 5.8)** | **3.2 (1.3, 5.1)** |
|  | % Trees | **3.1 (1.4, 4.7)** | **3.3 (1.6, 5.0)** | **2.8 (1.1, 4.5)** |
|  | % Grass | 0.6 (-0.7, 1.9) | 0.7 (-0.6, 2.0) | 0.4 (-0.9, 1.7) |
|  | % Other | **1.1 (0.1, 2.2)** | **1.1 (0.0, 2.1)** | 0.8 (-0.2, 1.9) |
| Behavioral Domain | % Total greenspace | **3.9 (1.5, 6.2)** | **4.0 (1.5, 6.6)** | **3.4 (0.9, 6.0)** |
|  | % Trees | **3.4 (1.2, 5.6)** | **3.6 (1.3, 5.9)** | **3.1 (0.8, 5.4)** |
|  | % Grass | 0.3 (-1.4, 2.0) | 0.4 (-1.4, 2.1) | 0.1 (-1.6, 1.8) |
|  | % Other | 1.2 (-0.2, 2.6) | 1.1 (-0.3, 2.4) | 0.9 (-0.5, 2.2) |
| Biomedical Domain | % Total greenspace | **4.7 (1.8, 7.7)** | **5.3 (2.2, 8.3)** | **4.4 (1.4, 7.5)** |
|  | % Trees | **4.0 (1.2, 6.7)** | **4.4 (1.6, 7.2)** | **3.8 (1.0, 6.5)** |
|  | % Grass | 0.4 (-1.7, 2.5) | 0.6 (-1.5, 2.8) | 0.2 (-1.9, 2.4) |
|  | % Other | **2.1 (0.4, 3.8)** | **2.1 (0.4, 3.8)** | **1.8 (0.2, 3.5)** |

NDVI: Normalized Difference Vegetation Index; SES: socioeconomic status

Model 1. Adjusted for NDVI

Model 2. Adjusted for NDVI + age and SES index score (incorporating both individual- and neighborhood-level metrics)

Model 3. Model 2 + race and ethnicity

All models corrected for potential clustering of individuals within census tracts.

We included the three greenspace components (% trees, % grass, % other greenspace) in the same model.

Bold font indicates results that are statistically significant (95% CI excludes the null).
